# Accurate mandibular canal segmentation of dental CBCT using a two-stage 3D-UNet based segmentation framework

**DOI:** 10.1186/s12903-023-03279-2

**Published:** 2023-08-10

**Authors:** Xi Lin, Weini Xin, Jingna Huang, Yang Jing, Pengfei Liu, Jingdan Han, Jie Ji

**Affiliations:** 1 Clinic of Stomatology of the Shantou University Medical College, No. 22, Xinling Road, Shantou, Guangdong China; 2 Department of Stomatology of Shantou University Medical College, No. 22, Xinling Road, Shantou, Guangddong China; 3 Huiying Medical Technology Co., Ltd, Room A206, B2, Dongsheng Science and Technology Park, Haidian District, Beijing, China; 4 Network and Information Center, Shantou University, No. 243, University Road, Shantou, Guangdong China

**Keywords:** Artificial intelligence, Dental radiology, Cone-beam computerized tomography, Inferior alveolar nerve

## Abstract

**Objectives:**

The objective of this study is to develop a deep learning (DL) model for fast and accurate mandibular canal (MC) segmentation on cone beam computed tomography (CBCT).

**Methods:**

A total of 220 CBCT scans from dentate subjects needing oral surgery were used in this study. The segmentation ground truth is annotated and reviewed by two senior dentists. All patients were randomly splitted into a training dataset (n = 132), a validation dataset (n = 44) and a test dataset (n = 44). We proposed a two-stage 3D-UNet based segmentation framework for automated MC segmentation on CBCT. The Dice Similarity Coefficient (DSC) and 95% Hausdorff Distance (95% HD) were used as the evaluation metrics for the segmentation model.

**Results:**

The two-stage 3D-UNet model successfully segmented the MC on CBCT images. In the test dataset, the mean DSC was 0.875 ± 0.045 and the mean 95% HD was 0.442 ± 0.379.

**Conclusions:**

This automatic DL method might aid in the detection of MC and assist dental practitioners to set up treatment plans for oral surgery evolved MC.

## Background

The MC is the most closely related to teeth among the bone dense canals containing blood vessels and nerves. It contains inferior alveolar nerve, artery and vein. Accurate segmentation of the mandible canal from CBCT is an important step for building a personalized 3D digital mandibular model for surgical procedures involving the posterior mandible, such as implant surgery, the removal of mandibular wisdom teeth, sagittal split osteotomy and cyst removal [1–6]. The MC injury may result in (semi)-permanent numbness and paresthesias in the innervated structures, such as lips, jaws, teeth, tongue, mucous membranes, gums [3, 6–9].

CBCT is widely used in oral clinic to help diagnose oral hard tissue diseases. However, teeth, tooth fillings, and dental braces in orthodontic treatment and metal implants in orthognathic treatment are high attenuation materials which cause high noise and low contrast in visual impressions of CBCT images. Specifically, weak and false edges in parts of condyles and teeth often appear in the CBCT images. Furthermore, it is difficult to identify the boundaries of the MC since the dental braces and metal implants negatively affect the image quality in CBCT, and there are individual differences in patients and devices [10]. Although manual analysis can still maintain high accuracy in the daily single-digit case analysis, the energy consumption of analysts is also obvious, and for inexperienced doctors, misjudgment may occur.

Previous methods usually conduct manual segmentation of the MC which takes huge amount of time to reconstruct 3D mandible models. And there are several semi-automated CBCT-guided planning software tools that support 3D MC visualization. However, the semi-automatic methods cannot achieve high precision and simple usability [2, 11–12]. Accurate segmentation of the MC is an important step for building a 3D mandibular model for surgical procedures involving the posterior mandible, such as implant surgery, the removal of mandibular wisdom teeth, sagittal split osteotomy and cyst removal. However, both manual segmentation and semi-automatic software segmentation have some problems, The main challenges in MC segmentation are as follows:

1. high noise and low contrast in CBCT images,
2. inaccurate density in CBCT images [13–14],
3. to identify the boundaries of the MC since the dental braces and metal implants negatively affect the image quality in CBCT [2],
4. anatomical variability among individuals [15].

The diagnosis from these CBCT results made by primary doctors is easily affected by inexperience. Therefore, there is a great demand for a rapid, accurate, and automatic segmentation method for MC, to eliminate the misdiagnosis caused by the above difficulties as much as possible.

Recently, deep convolutional networks have now become the technique of choice in computer vision. DL has been widely used in medical image computing. The most successful type of models for image analysis to date are convolutional neural networks(CNNs). CNNs contain many layers that transform their input with convolution filters of a small extent. CNNs are widely used in medical image classification, segmentation and other fields [16–17]. Base on two-stage segmentation framework with 3D-UNet, the objective of this study is to develop a deep learning model for fast and accurate mandibular canal segmentation on CBCT.

## Materials and methods

### Ethics approval and consent to participate

The experimental protocol was established, according to the ethical guidelines of the Helsinki Declaration and was approved by the Human Ethics Committee of the Shantou University Medical College (SUMC), Ethical Approval ID:SUMC-2022-085. Informed consent was obtained from all subjects and/or their legal guardians. This study had a non-interventional retrospective design, and there was no human experiment or use of human tissue samples. All the data were analyzed anonymously.

### Patients and dataset

In this study, dental records (including images) of 220 patients undergoing CBCT for oral surgery between June, 2021 and March, 2022 at the Clinic of Stomatology of the SUMC were used. Patients presenting previous surgical history or diseases of the oral, dysplasia mandible, the bone of mandible degenerates obviously and maxillofacial region were excluded. There were 136 (61.82%) females and 84(38.18%) males, aged 10–66 years, with an average age of 36.93 ± 13.77 years.We randomly sampled 132 (60%) of the CBCT scans for model training, 44 (20%) for model validation, and the remaining 44 (20%) were used as the testing set. All the data were annotated and reviewed using ITK-SNAP 3.8.0 software. Specifically, a senior dentist was responsible for delineating the MC, and another senior doctor was responsible for reviewing. If the opinions of the two dentists disagree, the final annotation results was determined after consultation.

**Fig. 1:**
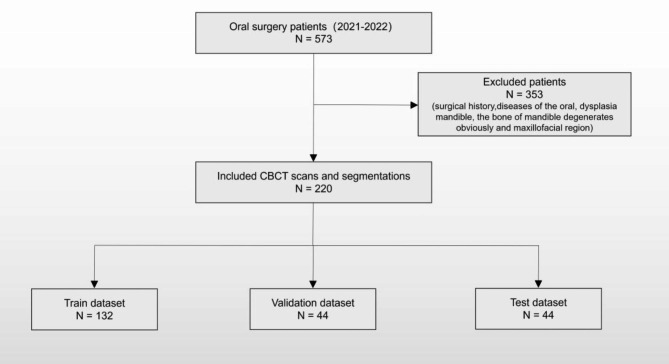
Flowchart of patient admission and discharge

The complete flowchart of the data collection process is shown in Fig. 1.

CBCT scans were performed by using a 3D Imaging Systems (Carestream Dental Co.) with 4 mA, 90 kV, a 8-s exposure time, per slice thickness of 180 μm, and a voxel of 180 μm× 180 μm × 180 μm.

### Data preprocessing

In data processing, we conducted the same procedure for all the data. Firstly, we clipped the intensity values of each scan to [-1000, 1945] to reduce the effect of extreme values, and then normalized truncated voxel values by subtracting its mean 307.49 and dividing by its standard deviation 195.61 [18–19].

### The two-stage 3D-UNet Architecture

**Fig. 2:**
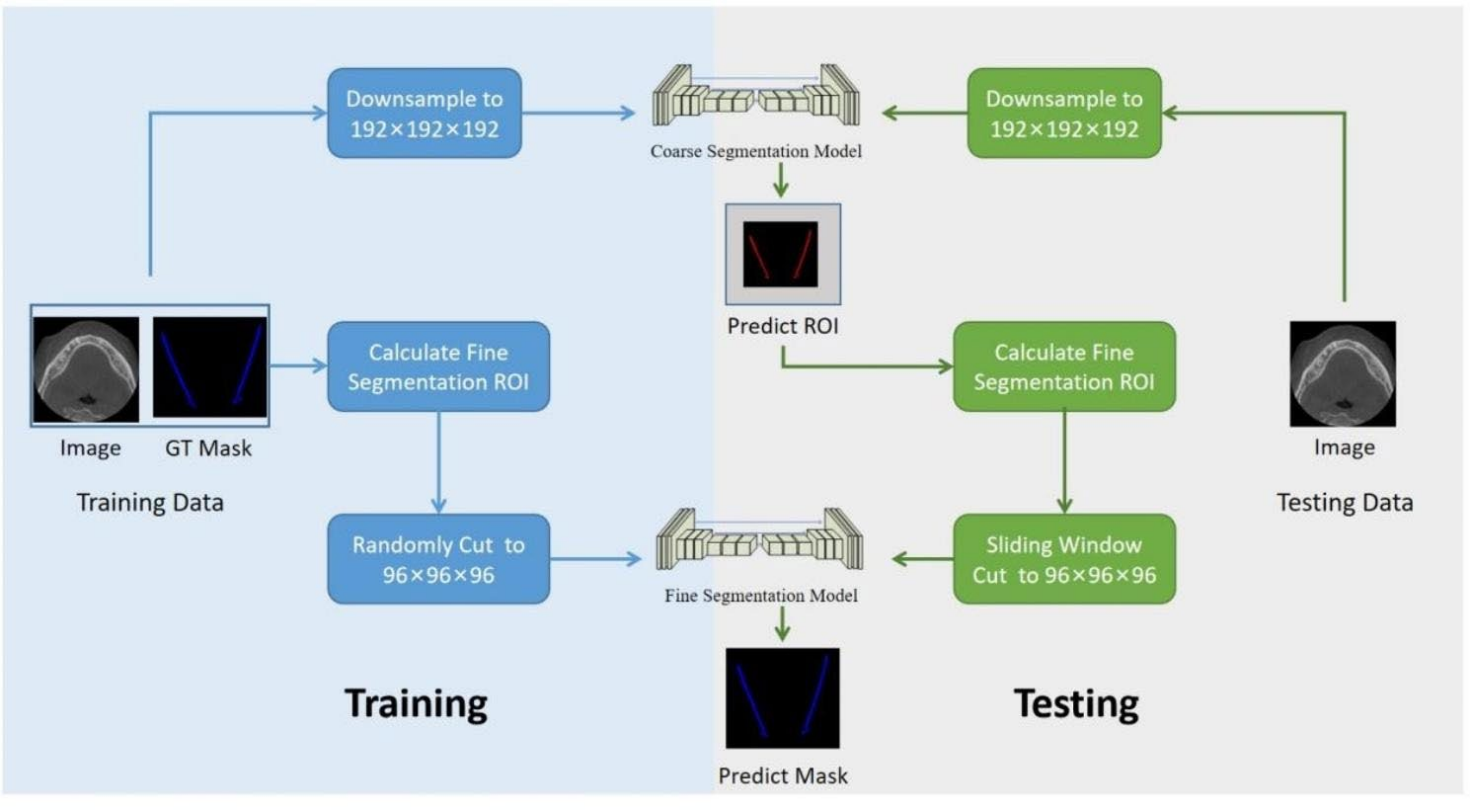
Overall model architecture

We adopted a two-stage 3D-UNet structure as the backbone of our segmentation model. A graphical illustration of the model is shown in Fig. 2 [20].

**Fig. 3:**
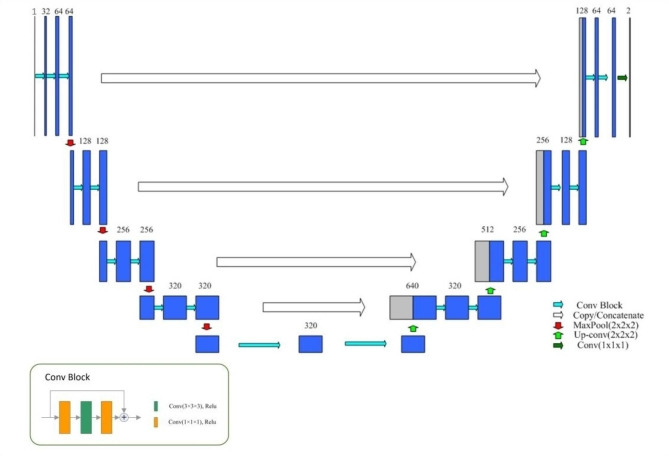
3D-UNet model structure

Small scaled MC structure is difficult to be recognized in the original image due to large uncertainty. In this paper, we adopted a two-stage approach to alleviate the above issue. This framework applies a coarse segmentation model and a fine segmentation model in sequence. Both models adopted 3D-UNet structure, which consists of encoder, decoder, dense skip pathway, and deep supervision [21]. The encoder generates high-dimensional features. The decoder realizes feature fusion and recovers the segmentation result. Dense connection realizes feature reuse through skip connection to enhance feature learning, the skip connections between encoder and decoder are also added to keep more low-level details for better segmentation. The deep supervised structure can accelerate the convergence of the network. In order to improve the gradient flow of the model, we replaced the convolution with the form of Bottleneck [22]. The network architecture is shown in Fig. 3.

The models were trained separately and independently for each stage. For the coarse segmentation model, we re-scaled the image to 192 × 192 × 192 as input. For the fine segmentation model, the minimum bounding boxes containing the ground-truth annotations were extracted as input. Due to the limitation of GPU memory, we randomly crop patches of size 96 × 96 × 96 of the ROI as inputs. The model that performs best on the validation dataset is chosen as the final model.

In prediction phase, the coarse segmentation model was used to localize the regions of interest. The minimum bounding box of the coarse segmentation result is expanded by 5 voxels in all 3 directions and is used as the input for the fine model. The sliding window operation was used for prediction, and the sliding window step size was 48 voxels in all directions. We took the union of the segmentation results for the overlapped regions.

Our work was implemented with Pytorch 1.10.0 DL framework. And the models were trained on a NVIDIA Tesla V100 GPU.

### Training process

The same data augmentation method was applied in both stages, which includes flipping, rotating, scaling, and gamma transforming. In this article, we randomly flip the data, and the rotation angles in all directions range between [-30, 30]. The scaling factor ranges from [0.85, 1.25], the gamma transform takes values between [0.7, 1.5]. All data augmentations were only used in the training stage.

In segmentation tasks, dice loss or cross-entropy loss is usually used. Dice Loss is a loss function based on the DSC. The cross entropy loss is used to evaluate the difference between the predicted category and the true category for each pixel. The specific forms of dice loss and cross-entropy loss are as follows:

$${L}_{dice}=1 - \frac{2{\sum }_{i}^{N}{p}_{i}{g}_{i}}{ {\sum }_{i}^{N}{p}_{i}^{2} +{\sum }_{i}^{N}{g}_{i}^{2}}$$

$${L}_{ce}=-\sum _{c=1}^{M}{y}_{c}log\left({p}_{c}\right)$$

In the dice loss function, calculating the sum of $N$voxels, of the predicted binary segmentation volume${p}_{i}\in P$ and the ground truth binary volume${ g}_{i}\in$ G. In the cross-entropy loss function, $M$ represents the number of categories where M takes 2, ${y}_{c}$ represents the ground truth label value which takes 0 or 1. If the category and the sample category are the same, ${y}_{c}$ takes 1, otherwise 0, and ${p}_{c}$ represents the probability that the predicted sample belongs to $c$.

In this paper, we used a hybrid loss combining cross-entropy loss and dice loss, the specific form is as follows:

$$L={\omega }_{dice}{L}_{dice}+{\omega }_{cross}{L}_{ce}$$

where ${\omega }_{dice}$ is the weight of the dice loss and ${\omega }_{cross}$ is the weight of the cross-entropy loss, in this study both ${\omega }_{dice}$ and ${\omega }_{dice}$ were set to 0.5.

The SGD optimizer was used along with a momentum of 0.95. The learning rate varies as the epoch decreases according to the following formula:

$$lr=initial\_lr\times {\left(1-epoch/max\_epochs\right)}^{exponent}$$

where the $initial\_lr$ was 0.01, the $max\_epochs$ was 1000, and the $exponent$ was 0.9.

To make the segmentation less sensitive to small false positive regions, a post-processing procedure is adopted. Only two largest regions in the segmentation results were kept as the final segmentation of the left and right MC.

### Evaluation Metrics

To measure the performance of the DL model, Dice Similarity Coefficient (DSC) and 95% Hausdorff Distance (95% HD) were utilized in this study.

DSC is a set similarity measure, which measures the volumetric overlap between the predicted segmentation and the ground-truth annotation. The value range is [0,1]. The best value of the segmentation result is 1, and the worst value is 0, the calculation formula is in Sect. 2.4.

The Hausdorff Distance is a measure of the degree of similarity between two sets of points. It is commonly used in image segmentation tasks that are sensitive to the segmentation boundary. 95% HD is based on the calculation of the 95th percentile of the distances between boundary points in two sets of points, to eliminate the influence of very small subsets of inaccurate segmentations on the overall segmentation quality. The calculation formula is as follows:

$$\eqalign{&95\% HD \cr & =max\left\{{maxd(t, S(P\left)\right)}_{P95}, \right. \cr & \left. {maxd(p, S(T\left)\right)}_{P95}\left|t \epsilon S\right(T), p \epsilon S(P)\right\}}$$

Where $d(b, B)={min}_{b \epsilon B}\left\{\parallel {a-b}_{2}\parallel \right\}$. The $t$ represents the coordinates of the ground truth canal voxel, the $p re$presents the coordinates of the model predicted canal voxel, T is the set of ground truth canal voxel coordinates, $P$ is the set of predicted canal voxel coordinates, and $S(\bullet )$ represents an operation that extracts the surface voxels of a set of voxels.

## Results

**Fig. 4:**
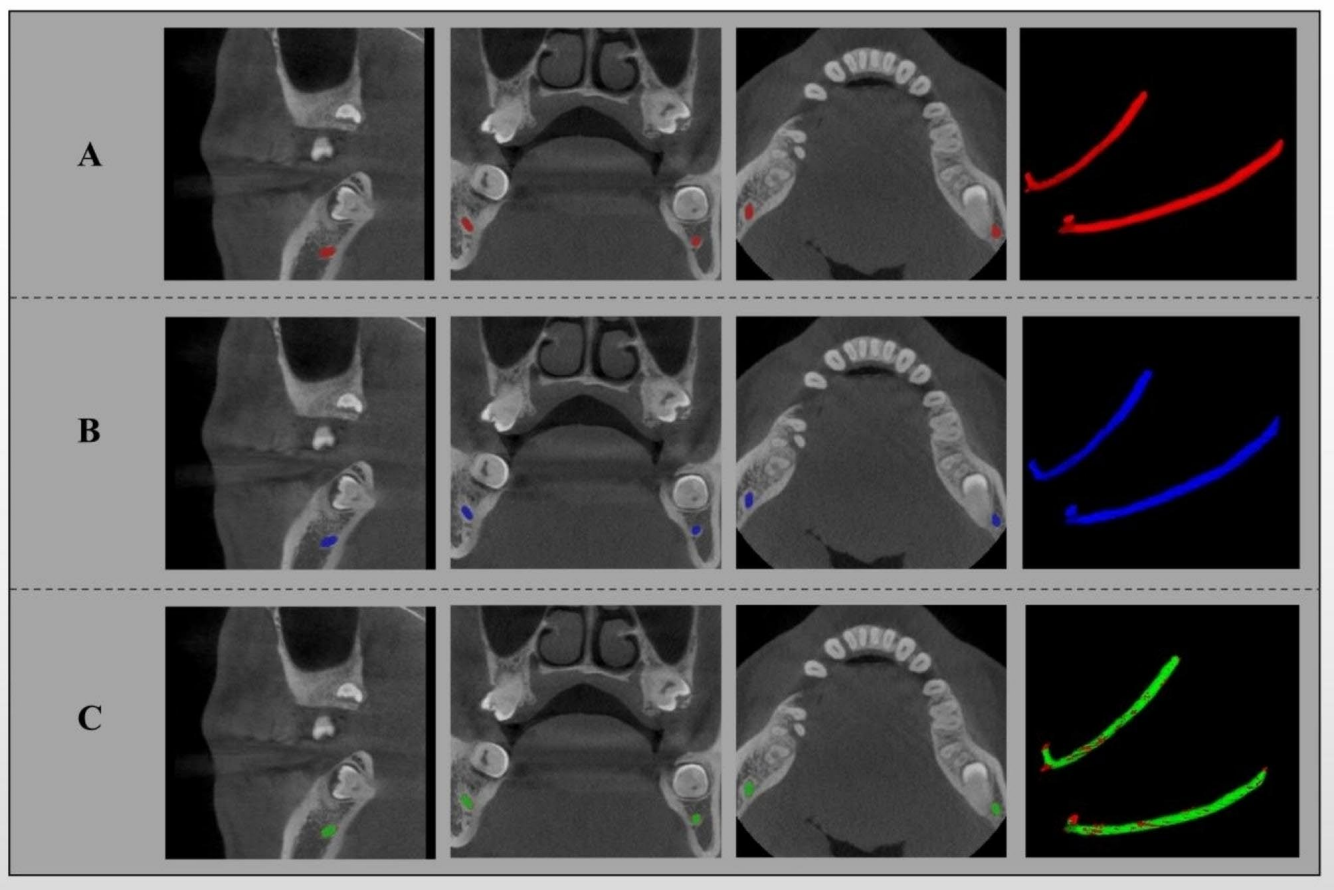
Visualization of our output. (**A**) segmentation result (red); (**B**) manual ground-truth (blue); (**C**) overlapped (green) and non-overlapped(red) regions of A and B. Sagittal (first column), coronal (second column), axial (third column), 3D models of the MC(fourth column)

The segmentation results are shown in Table 1, and several segmentation samples are shown in Fig. 4.

**Table 1:** Results of the two-stage 3D-UNet segmentation model

Model	Data grouping	DSC	95% HD (mm)
Coarse segmentation	Train dataset	0.847 ± 0.038	0.508 ± 0.564
	Validation dataset	0.860 ± 0.040	0.630 ± 0.585
	Test dataset	0.827 ± 0.057	1.027 ± 1.422
Fine segmentation	Train dataset	0.872 ± 0.028	0.417 ± 0.170
	Validation dataset	0.884 ± 0.023	0.352 ± 0.104
	Test dataset	0.875 ± 0.045	0.442 ± 0.379

From the above table, we can find that in the training set, validation set, test set, the fine segmentation model is better than the coarse segmentation model in terms of both DSC and 95%HD. For the DSC, the overall performance of the fine segmentation model is 2 to 5% points higher than that of the coarse segmentation model. For the 95%HD, compared with the coarse segmentation model, the similarity predicted by the fine segmentation model is much more accurate, especially in the test set, the value of 95%HD is reduced by 0.585.

Meanwhile, we calculate the number of parameters of the models and the inference times. The coarse segmentation model has 29.7 M parameters and the fine segmentation model includes 29.1 M. It takes about 1.54s to inference a 192 × 192 × 192 patch for the coarse segmentation model. The fine segmentation model takes 0.37s to inference a 96 × 112 × 224 patch.

## Discussion

DL has been widely used in the field of medical image segmentation [23].In many tasks, end-to-end deep neural networks have been shown to be superior to traditional methods [24–25]. Regardless of the limitation of GPU memory and computing power, compared to a single 3D-UNet model, the two-stage model can lead to better performance for certain tasks, such as segmentation of sclerosis lesion, liver, spleen and pancreas [26–27].

There are several difficulties in CBCT image segmentation. For example, the small-scaled targets, unclear boundary, high noises and low contrast in CBCT images, etc. [28.–29]. Although the segmentation of the MC faces many challenges, our finding results represent a great improvement over many previous related studies. Our results for the segmentation of MC (DSC of 0.884, 95% HD of 0.352, in the validation set) are significantly superior to those reported by Lahoud P et al. (DSC of 0.774, 95% HD of 0.705) and Jaskari J et al. (DSC of 0.575, 95% HD of 1.39) [2, 15].

In this study, we adopted a two-stage coarse-to-fine 3D-UNet framework to segment the medical images of the inferior alveolar nerve canal, it can verify the advantages of using DL algorithms in the high-precision reconstruction of 3D bony anatomical structures. The two-stage 3D-UNet is likely to be more suitable for the small segmentation targets than the single 3D U-Net. The coarse model is used to localize the MC, and the fine segmentation model is used to perform a fine-grained segmentation. From the result of this study, the segmentation of mandibular canal based on the two-stage 3D-UNet can meet the needs of dentate subjects needing oral surgery.

According to our results, the MC segmentation based on DL is likely to play a more active role in the future However, the clinical deployment of DL models is closely related to model size and inference time. In this study, the number of parameters of the coarse segmentation model has 29.7 M and the fine segmentation model includes 29.1 M. In the following research, we plan to compress and solidify the model to make the model occupy less storage and more convenient for terminal deployment. In addition, a 192 × 192 × 192 patch for the coarse segmentation model takes about 1.54s, and the fine segmentation model takes 0.37s to predict the MC in our study. This makes the prediction time for a case likely to exceed ten seconds or more. In the follow-up research, we will optimize related algorithms to shorten the prediction time of a single patch.

This study still has several limitations. This research is a single-center study, and the generalization ability of the segmentation model has not been verified in other cohorts. In addition, more data is needed in order to verify the reliability of the model under multiple variables (age, gender, and ethnicity). And there is still a lot of room for improvement in the efficiency of model inference and the optimization of the model capacity. In the future, we will focus on solving above problems.

To conclude, the findings of this study demonstrate the potential of DL algorithms in automated MC segmentation. Automatic segmentation algorithm will play a positive role in the planning of operations involving the MC.

## Conclusion

The MC segmentation is a relatively complex task due to many challenges. We developed a two-stage 3D-UNet deep neural network for accurate segmentation of the MC, and the segmentation results were greatly improved compared with previous studies. We believe that the DL model of the MC segmentation will bring some positive changes to clinical oral surgery planning.

## Data Availability

All data generated or analysed during this study are included in this published article.

## List of Abbreviations

DL: Deep learning
MC: Mandibular canal
CBCT: Cone beam computed tomography
DSC: Dice Similarity Coefficient
95% HD: 95% Hausdorff Distance
CNNs: Convolutional neural networks
